# Klotho in pregnancy and intrauterine development—potential clinical implications: a review from the European Renal Association CKD-MBD Working Group

**DOI:** 10.1093/ndt/gfae066

**Published:** 2024-03-14

**Authors:** Mehmet Kanbay, Ali Mutlu, Cicek N Bakir, Ibrahim B Peltek, Ata A Canbaz, Juan Miguel Díaz Tocados, Mathias Haarhaus

**Affiliations:** Department of Medicine, Division of Nephrology, Koc University School of Medicine, Istanbul, Turkey; Department of Medicine, Koc University School of Medicine, Istanbul, Turkey; Department of Medicine, Koc University School of Medicine, Istanbul, Turkey; Department of Medicine, Koc University School of Medicine, Istanbul, Turkey; Department of Medicine, Koc University School of Medicine, Istanbul, Turkey; Vascular and Renal Translational Research Group, Biomedical Research Institute of Lleida, Dr Pifarré Foundation (IRBLleida), Lleida, Spain; Division of Renal Medicine, Department of Clinical Science, Intervention and Technology, Karolinska University Hospital, Karolinska Institutet, Stockholm, Sweden; Diaverum AB, Malmö, Sweden

**Keywords:** Klotho, organogenesis, placenta, preeclampsia

## Abstract

Intrauterine development is crucial for life-long health; therefore, elucidation of its key regulators is of interest for their potential prognostic and therapeutic implications. Originally described as a membrane-bound anti-aging protein, Klotho has evolved as a regulator of numerous functions in different organ systems. Circulating Klotho is generated by alternative splicing or active shedding from cell membranes. Recently, Klotho was identified as a regulator of placental function, and while Klotho does not cross the placental barrier, increased levels of circulating α-Klotho have been identified in umbilical cord blood compared with maternal blood, indicating that Klotho may also play a role in intrauterine development. In this narrative review, we discuss novel insights into the specific functions of the Klotho proteins in the placenta and in intrauterine development, while summarizing up-to-date knowledge about their structures and functions. Klotho plays a role in stem cell functioning, organogenesis and haematopoiesis. Low circulating maternal and foetal levels of Klotho are associated with preeclampsia, intrauterine growth restriction, and an increased perinatal risk for newborns, indicating a potential use of Klotho as biomarker and therapeutic target. Experimental administration of Klotho protein indicates a neuro- and nephroprotective potential, suggesting a possible future role of Klotho as a therapeutic agent. However, the use of Klotho as intervention during pregnancy is as yet unproven. Here, we summarize novel evidence, suggesting Klotho as a key regulator for healthy pregnancies and intrauterine development with promising potential for clinical use.

## INTRODUCTION

The intrauterine period forms the basis for lifelong health and well-being. The complex interplay of genetic and environmental factors at this critical stage can influence susceptibility of an individual to various diseases later in life. Klotho, a protein initially identified in the kidney, has emerged as an important regulator of healthy metabolism and longevity with potential clinical implications. While initial research focused on its anti-aging properties [1], recent studies have claimed that Klotho also plays a role in intrauterine life and organogenesis [2].

Klotho was discovered in mice as a protein responsible for suppression of aging [1]. Several Klotho proteins have been identified. These proteins are all single-pass transmembrane proteins, including α-, β- and γ-Klotho. α-Klotho and β-Klotho play crucial roles as integral constituents of receptor complexes for endocrine fibroblast growth factors (FGFs). They are necessary for the high-affinity interaction between FGFs and their respective FGF receptors (FGFRs). Together, these proteins form a distinctive endocrine system that governs a variety of metabolic processes in mammals [3]. The role of Klotho in embryogenesis and foetal development is still not fully understood, but novel research indicates that Klotho is involved in organogenesis and intrauterine growth (Table 1). Elucidating the role of Klotho in the early human development has the potential to guide interventions that promote lifelong health. In this narrative review, we aim to summarize the evolving knowledge of the molecular structure and functions of Klotho with a specific focus on its expression and regulatory mechanisms during pregnancy and intrauterine development, exploring its potential impact on foetal and maternal health, intrauterine metabolism, and its utility as a biomarker for predictive and diagnostic purposes, ending in a discussion of potential therapeutic roles and paths for future research in the area of Klotho in intrauterine life.

## MOLECULAR STRUCTURE AND FUNCTION OF KLOTHO

Klotho comprises three subfamilies, α-Klotho, β-Klotho and γ-Klotho [3]. When the article does not specify a subfamily, the term ‘Klotho’ typically refers to the α-Klotho subfamily.

α-Klotho is a type I single pass 130 kDa transmembrane protein comprising of a large extracellular domain with two internal repeats (KL1, KL2), a transmembrane domain and a small cytoplasmic tail [4]. Two primary forms of α-Klotho exist, the full-length membrane form and a soluble circulating form in blood, urine and cerebrospinal fluid, generated through alternative splicing or cleavage of the extracellular domain by a disintegrin and metalloproteinase domain-containing protein (ADAM)10 and ADAM17 [3]. Klotho binds to the FGFR, forms the Klotho–FGFR complex, and binds to Fibroblast Growth Factor 23 (FGF23), activating downward signalling [5]. Soluble Klotho can function as a coreceptor of FGF23 or, independent of FGF23, as a circulating hormone, local autocrine/paracrine factor or an enzyme regulating ion channels [4].

α-Klotho is encoded by the *Klotho* (*KL*) gene on chromosome 13 in humans, which is syntenic to the locus of KL in mice on chromosome 5 and KL in rats on chromosome 12 between *PDS5B* and *STARD13* [6]. β-Klotho is transcribed by the *KLB* gene, located on chromosome 4 in humans [7] and the gene transcribing γ-Klotho is located on chromosome 15 [8]. β-Klotho is a type I transmembrane protein expressed predominantly in adipose tissue, liver and brain, functioning as a mediator for FGF21 and FGF19 proteins on metabolic regulation, glucose uptake, bile acid synthesis, alcohol consumption and fatty acid metabolism synthesis [9]. The functions of γ-Klotho are as yet widely unknown, but it is highly expressed in the lens of the eye and in concordance with the other Klotho proteins it may be associated with aging processes.

## EXPRESSION AND REGULATION OF KLOTHO

α-Klotho is the most extensively studied among the Klotho proteins and its biological functions are better known than those of other Klotho proteins [6]. α-Klotho is predominantly expressed on the cell membrane of distal renal tubules, the parathyroid gland and the choroid plexus of the brain; the expression is modulated by several physiological and pathological states [5]. α-Klotho expression decreases in acute kidney injury and many processes involved in the development of chronic kidney diseases, including kidney fibrosis and diabetic nephropathy [10]. Several pro-inflammatory transcription factors, epigenetic and non-epigenetic regulators have a potential to downregulate α-Klotho, including angiotensin-II, nuclear factor κ-light-chain enhancer of activated B cells (NF-κB) signalling, matrix metalloproteinases, reactive oxygen species, miRNAs, and DNA methylation and acetylation [10]. Conversely, vitamin-D responsive elements, epidermal-growth factor, peroxisome proliferator-activated receptor (PPAR)-γ signalling pathway, aerobic exercise and medications including losartan, statins, fosinopril and erythropoietin upregulate the expression of α-Klotho [11–15].

## EMBRYOGENESIS, KLOTHO AND SIGNALLING PATHWAYS

The role of Klotho in pregnancy and intrauterine development is a fascinating novel area of research. In a study that used a model of kidney development in zebrafish, demonstrating evolutionary conservation of the FGF23–Klotho axis, α-Klotho could be detected as early as 24 h postfertilization in the brain, pancreas and the distal pronephros [16]. It is evident that Klotho proteins exhibit numerous interactions with multiple signalling pathways that play pivotal roles in placental function and embryonic development.

### Wnt signalling pathway

Klotho governs the process of stem cell aging by controlling the Wnt (Wingless-related integration site) signalling pathway [17]. Wnt/β-catenin plays a fundamental role in embryogenesis, cell differentiation, proliferation and apoptosis. Abnormal Wnt signalling leads to various diseases, including cancer, lung diseases, cardiovascular diseases and neurodegenerative disorders [18]. Klotho inhibits the activation of the Wnt signalling pathway, and Klotho deficiency causes aging of stem cells by persistent activation of the Wnt signalling pathway and transcription of cytokines [19]. In an *in vitro* oocyte maturation study, treatment of porcine oocytes with 5 pg/mL Klotho increased cumulus cell expansion, blastocyst formation rates and the total cell number of blastocysts [20]. In addition, the administration of Klotho protein led to an increase in cellular ATP levels and bolstered antioxidant activities [20]. These findings demonstrate the significance of Klotho in modulating the Wnt signalling pathway, suggesting its potential importance in oocyte maturation and embryogenesis [20].

### Growth hormone/insulin-like growth factor-1 signalling pathway

Insulin-like growth factor-1 (IGF1) has a direct effect on foetal cell proliferation, differentiation and apoptosis, and there is a well-established association between the serum concentration of IGF1 and foetal growth and length [21]. While the intrauterine effect of IGF1 is independent of growth hormone (GH) [22], IGF1 influences postnatal growth and sexual maturation during puberty as mediator of GH, but its serum concentration decreases sharply after puberty [22]. In later stages of life, circulating IGF1 levels correlate positively with shorter life span [23]. Klotho exerts an inhibitory effect on IGF1 signalling pathways and this effect has been proposed as one of the anti-aging mechanisms of Klotho [24]. α-Klotho decreases oxidative stress and counteracts senescence in the placenta by suppressing the IGF1 signalling pathway. The interaction of Klotho, GH and IGF1 is complex. Klotho and IGF1 increase in response to GH treatment in normal subjects and patients with chronic kidney disease [25]. Klotho induces and IGF1 inhibits pituitary GH secretion, while IGF1 induces Klotho secretion [26]. In spite of increased levels of circulating Klotho and IGF1 during intrauterine development, interactions between Klotho and IGF1 during prenatal life have not yet been investigated.

Both α-Klotho and β-Klotho play a role in gestational diabetes mellitus by inducing insulin resistance [27]. In an *in vitro* model of gestational diabetes mellitus, Klotho was found to be involved in the insulin resistance of trophoblast cells, partially mediated through the IGF1/phosphatidylinositol 3-kinase (PI3K)/Akt/mammalian target of rapamycin (mTOR) pathway [27]. The physiological role of this pro-diabetic effect of Klotho in gestational diabetes mellitus is as yet unclear. A possible protective effect from cellular overnutrition, especially lipid overloading, has been proposed [28].

### Hypoxia-inducible factor pathway

Hypoxia-inducible factors (HIF) are heterodimeric transcription factors and primary regulators of molecular response to hypoxia [29]. HIF isoforms are expressed early in embryonic development in response to physiological hypoxia [29]. HIF1α expression plays a fundamental role in tissue development and functioning of stem cells. Klotho deficiency causes increased expression of HIF1α and Klotho overexpression reduces the HIF1α levels [29]. HIF is induced in early placental development and is one of the key factors that control placental function. HIF is involved in preeclampsia, placental aging and preterm labour [29]. While Klotho is also involved in preeclampsia [30], placental aging and preterm delivery [30], to our knowledge, a possible interaction of Klotho with HIF in regulation of placental function, preeclampsia or preterm delivery has not yet been studied.

## ROLE OF KLOTHO IN PLACENTAL AND INTRAUTERINE METABOLISM

### Cholesterol

β-Klotho is expressed in adult and embryonic tissues; Kobayashi *et al*. demonstrated that β-Klotho expression in the yolk sac impacts on foetal growth and metabolism [31]. β-Klotho is crucial for foeto-maternal cholesterol transport, highlighting the importance of the β-Klotho–FGF15 axis. Embryos lacking β-Klotho were smaller post-implantation, persisting into postnatal stages, indicating long-term consequences of intrauterine growth restriction (IUGR) [31].

Ding *et al*. have shown that the role of β-Klotho in adipose tissue is crucial in facilitating the short-term effects of FGF21 on insulin sensitivity and glucose uptake in mice. They concluded that β-Klotho is required for the effects of FGF21 on body composition, lipid homeostasis and carbohydrate homeostasis, including its insulin-sensitizing actions [32]. The metabolic effects of FGF21 have been demonstrated to be absent in lipodystrophic mice, offering additional evidence that FGF21 exerts one of its actions on adipose tissue [33].

### Mineral homeostasis

The development of the foetus during pregnancy requires an excess of calcium and phosphate, compared with the mother. Active transportation of calcium and phosphate across the placenta to the foetal circulation contributes to higher circulating concentrations of these minerals in the foetus than in the mother [34]. Elevated levels of soluble α-Klotho have been described in human cord blood and an elevated gene expression of KL was found in the human placenta [35]. FGF23 is an endocrine hormone released by bone tissue, and its best known function is to control phosphate homeostasis by targeting the kidney and parathyroid glands to promote increased phosphaturia and inhibition of parathyroid hormone production/secretion, respectively [16]. Klotho is a coreceptor which increases the affinity of FGF23 to FGFRs [16]. Klotho and FGF23 are associated with placental function, including placental calcium and phosphorus transport [16].

The transportation of calcium occurs primarily through the transient receptor potential cation channel subfamily V (TRPV5 and TRPV6), channels known to be activated by α-Klotho [36]. Inhibition of chorionic somatomammotropin hormone in sheep resulted in an IUGR phenotype with lower umbilical calcium uptake and lower expression of α-Klotho, FGFR1 and TRPV6 in the foetal component of the placentome [37].

Despite the pivotal role of FGF23/Klotho for postnatal phosphorus metabolism, FGF23 was not found to be essential for foetal phosphorus metabolism in a mouse study [38]. Intact FGF23 was not increased in Klotho deficient foetuses, and Klotho-deficient foetuses had normal bone length, morphology and mineralization; however, the mice became hyperphosphatemic 5 days after birth [38]. Godang *et al*. [35] described significantly higher levels of α-Klotho and sclerostin and significantly lower 25-hydroxyvitamin D [25(OH)D] levels in umbilical cord plasma at gestational week 30–32, compared with maternal levels, while they found no difference in FGF23 levels. However, only neonatal sclerostin levels were independently associated with neonatal total body mineral content (BMC), while no association was found with maternal sclerostin values or circulating α-Klotho, FGF23 or 25(OH)D levels on either the foetal or maternal side of the placenta [35]. Circulating calcium and phosphate levels were only determined in the mothers and were not associated with neonatal BMC [35]. Contrary to the key functions of the FGF23/Klotho signalling on mineral metabolism in postnatal conditions, these studies indicate slight contributions of this pathway in the prenatal stage.

### Glucose

Klotho modulates glucose metabolism, and its expression is upregulated in the placentas of women with gestational diabetes mellitus (GDM) [27]. GDM increases the risk for placental dysplasia and trophoblast dysfunction, which are essential for normal foetal development [27]. High glucose impairs human chorionic trophoblast cell viability [27]. Interestingly, Klotho expression was stimulated by high glucose, and down-regulation of Klotho improved cell viability and insulin sensitivity by activating the IGF1, IGF1R and PI3K pathways. These results suggest Klotho's potential role in GDM pathology and reinforces Klotho's potential as a treatment target for GDM [27]. Additionally, it has been discovered that silencing Klotho in high glucose-treated cells increased IGF1R, p-IGF1R and IGF1 expression, and activates the IGF1/PI3K/Akt/mTOR signalling pathway, known to be associated with insulin resistance [39]. Overexpressing Klotho reversed these effects, aligning with Klotho's known function of reducing Akt phosphorylation to weaken insulin signal transduction [39].

## POTENTIAL ROLES OF KLOTHO IN HAEMATOPOIESIS

Haematopoiesis takes place throughout intrauterine development and continues into adulthood [40]. The hematopoietic stem cell (HSC) microenvironment maintains homeostasis by proliferation, differentiation and migration of progenitor cells in steady state and in emergency [41]. Dysfunction or depletion of stem cells and progenitor cells contributes to aging [42]. Kidneys are well known for their involvement in erythroid differentiation of HSCs [43]. Klotho knock-down mice demonstrated increased progenitor cell senescence, including a reduction in HSCs characterized by decreased c-kit+ sca-1+ cell lineage [44]. Similarly, Klotho-deficient mice have an overall reduction in the HSC pool size in the bone marrow, disturbed erythropoiesis and impaired migratory properties of bone marrow cells, which can be reversed by exogenous Klotho administration [45]. Moreover, the hematopoietic abnormalities were present in the foetal liver in Klotho deficient mice, indicating that the effect of Klotho in haematopoiesis is independent of the bone microenvironment and is essential in developmental stages [45]. Klotho expressed in kidneys and inorganic phosphate regulate HSC maintenance in bone marrow [46]. Thus, Klotho has a role in pre- and postnatal erythropoiesis, but its exact function requires further elucidation.

## ROLE OF KLOTHO IN ORGANOGENESIS

### Potential roles of Klotho in nephrogenesis

Ali *et al*. designed an animal model of hyperoxia-induced kidney injury in the neonatal period [2]. Rats exposed to hyperoxia for 21 days in the neonatal period had decreased kidney Klotho gene and protein expression. When the rats were administered Klotho for 21 days, renal antioxidant capacity was increased, demonstrated by increased renal catalase and manganese superoxide dismutase protein expressions. The administration of Klotho also improved the restricted renal perfusion profile, prevented hyperoxia-induced glomerulopathy and attenuated tubular injury [2].

In addition, Klotho was found to be associated with vascular endothelial growth factor (VEGF)-receptor 2 (VEGFR-2), regulating transient-receptor potential canonical Ca^2^^+^ channel 1 (TRPC-1) and maintaining endothelial integrity [47]. In this study, Kusaba *et al*. observed that VEGF-mediated internalization of the VEGFR-2/TRPC-1 complex was disrupted in Klotho-deficient endothelial cells, while surface TRPC-1 expression increased [47]. The association between Klotho and VEGF is essential in terms of understanding whether Klotho has a role in foetal renal development. Although the exact role of VEGF in foetal development is not clear, it stimulates kidney development, and it is present in the glomerulus and distal tubule of both the foetal and adult kidneys [48]. Further studies are needed to better understand the role of Klotho in nephrogenesis and its molecular mechanisms.

### Potential roles of Klotho in neurogenesis

Mouse brain Klotho expression is seen already in late embryonic development [49]. Klotho is expressed in the neurons throughout the brain, highest in the choroid plexus cells generating cerebrospinal fluid. In an animal study that measured Klotho expression during dentate development comparing wild-type and Klotho knockout mice, gross hippocampal development was not different until 7 weeks of age [49]. However, by 3 weeks of age, Klotho-deficient mice had a decreased number of proliferating cells in the subgranular zone. In contrast, 6-month-old Klotho overexpressing mice exhibited increased numbers of neural stem cells, increased proliferation and more immature neurons with enhanced dendritic arborization, demonstrating that Klotho impacts stem cell functioning and regulates post-natal neurogenesis as early as 3 weeks of age but not earlier [49]. In another study that investigates the effect of intermittent fasting on cognitive functioning, Klotho is required for *in vitro* hippocampal neurogenesis and is increased by intermittent fasting in animal models [50].

## KLOTHO IN FOETAL AND MATERNAL HEALTH

Small for gestational age (SGA) is defined as a birth weight below the 10th percentile for gestational age whereas foetal growth restriction or IUGR is a clinical term, which describes neonates who exhibit symptoms of malnutrition and restricted growth during gestation, regardless of their birth weight percentile [51]. IUGR is associated with increased perinatal morbidity and mortality. While IUGR can result in SGA, some SGA infants are constitutionally normal and do not have an increased risk. Placental insufficiency, preeclampsia and advanced maternal age are risk factors for IUGR SGA [52]. The role of Klotho in IUGR SGA is still a subject of debate. In the placenta, α-Klotho is mainly expressed in the syncytiotrophoblast [53]. Plasma levels of α-Klotho are higher in pregnant women than in non-pregnant women and increase progressively during pregnancy. However, placental gene expression and plasma concentrations of α-Klotho in women with preeclampsia and SGA neonates are lower than in women with uncomplicated pregnancies [54, 55]. Fan *et al*. [53] demonstrated a positive correlation between serum α-Klotho concentrations (maternal and umbilical cord) and birth weight in normal pregnancies and in women with preeclampsia, which was more prominent in maternal than in umbilical cord serum. Placental aging serves as a physiological mechanism for labour induction. Nevertheless, accelerated aging of the placenta due to lower Klotho expression may be involved in preeclampsia and/or IUGR [56]. Cecati *et al*. [57] described reduced levels of *Klotho* mRNA and protein and a decrease in telomere length in preeclamptic placental tissue compared with placentas from uncomplicated pregnancies.

In foetuses with IUGR, skeletal muscle undergoes significant changes in cellular pathways associated with tissue growth, injury response and metabolism. The activation of muscle β‐Klotho by circulating FGF21 could potentially contribute to the mediation of adaptive adjustments in IUGR, notably impacting a decrease in muscle growth by inhibiting mTOR signalling [58].

Although Klotho-deficient mice are viable with early aging like properties including arteriosclerosis and short life span [1], pigs carrying foetuses with a monoallelic knockout of the *KL* gene faced challenges in maintaining pregnancies to full term. The observed reduction in Klotho expression within the placenta is likely a key factor, contributing to the observed pregnancy losses [59]. The placentas of foetuses with a monoallelic knockout of the *Klotho* gene exhibited altered expression of genes associated with aging and apoptosis, accompanied by reduced levels of the Klotho protein [59]. Additionally, a reduction in Klotho expression may lead to increased oxidative stress by stimulating the IGF1 signalling pathway [60].

## KLOTHO AS A BIOMARKER IN PREGNANCY AND INTRAUTERINE DEVELOPMENT

The current understanding of Klotho's role in physiological and pathological states (Table 2) has led to the evaluation of its potential as a biomarker for healthy pregnancy and foetal health [61]. Klotho has been suggested as a diagnostic tool for several pregnancy-associated diseases, including intrahepatic cholestasis of pregnancy (ICP), preeclampsia and gestational diabetes [61]. However, existing clinical studies demonstrate varying results. A study comparing 23 participants with healthy pregnancies with 19 with severe preeclampsia showed that maternal α-Klotho levels <830 pg/mL predicted preeclampsia with 89.5% sensitivity (95% confidence interval 66.9–98.7) and 73.1% specificity (95% confidence interval 52.2–88.4) [53]. In another study, comparing 36 women with preeclampsia versus 28 healthy women and 10 with chronic hypertensive pregnancy, serum Klotho above 12.48 pg/mL identified the presence of preeclampsia with a sensitivity of 100% and specificity of 96% [61]. Miranda *et al*. found that maternal plasma α-Klotho levels were higher in women with uncomplicated pregnancy, while mothers who delivered SGA neonates shown decreased plasma Klotho concentration compared with those who delivered appropriate for gestational age neonates, regardless of the presence of preeclampsia [54]. Infants with bronchopulmonary dysplasia and pulmonary hypertension demonstrated lower umbilical cord plasma Klotho compared with healthy controls. [62]. Tayyar *et al*. found lower maternal β-Klotho levels only in mothers with repeated ICP, not in *de novo* ICP or in healthy controls [63].

**Table 1:** A highlight of the knowns and unknowns about Klotho and its clinical implications in pregnancy and intrauterine development.

Knowns	Unknowns
Klotho has a role in erythropoiesis [45]	The precise mechanisms through which Klotho influences hematopoiesis and its specific function in foetal erythropoiesis remain unclear
Klotho is associated with VEGFR-2, regulating TRPC-1 and maintaining endothelial integrity, highlighting a potential role in foetal renal development [47, 48]	The precise role of Klotho in nephrogenesis, its contribution to renal development and its potential interaction with VEGF are unclear
Klotho expression in mouse brain is evident as early as late embryonic development, and it is found in neurons throughout the brain, with the highest expression in choroid plexus cells [49]	The exact mechanisms through which Klotho influences stem cell functioning, regulates post-natal neurogenesis as early as 3 weeks of age, and its role in earlier stages of neurogenesis is unknown
In preeclampsia and SGA neonates, the maternal and umbilical cord plasma concentration of α-Klotho was found to be lower [54]	The exact mechanisms of how Klotho influences placental function, contributes to preeclampsia and affects foetal development, especially in the context of SGA neonates, are not fully understood
Lower levels of α-Klotho in both maternal and umbilical cord serum have been associated with severe preeclampsia, and ROC curve analysis suggested its potential as a predictive tool for preeclampsia [61]	Validation of Klotho's efficacy as a reliable biomarker, establishing standardized measurement techniques for accurate assessment, and understanding the impact of confounding variables including maternal age, gestational age and underlying health conditions need further research
Klotho has demonstrated a neuroprotective role in mice studies [49, 60]	The associations between Klotho and biochemical events in the perinatal period remain largely unexplored, making it an area of unknown effects and potential significance
Increasing Klotho levels plays a vital role in mitigating renal disease progression by inhibiting RAAS, reducing fibrosis and oxidative stress, and improving kidney structure and function including elevated eGFR and reduced serum creatinine levels [10]	The potential use of Klotho in premature infants to prevent decreased nephron endowment and address kidney injury due to hyperoxia-related oxidative stress needs further research in order to understand its feasibility and specific effects
Injection of Klotho into animal models has shown positive results and its potential in some areas such as neurodegenerative conditions, kidney health and Type 1 diabetes [24]	Translating promising results from animal studies to human applications of Klotho faces challenges, including the lack of a standardized measurement method, uncertainties about dosing, safety gaps and the absence of detailed pharmacokinetic data; addressing these issues requires further investigation to enhance our understanding of Klotho's therapeutic potential in humans

ROC, receiver operating characteristic; RAAS, renin–angiotensin–aldosterone system; eGFR, estimated glomerular filtration.

**Table 2:** Klotho: effects and regulation.

**Effects of high Klotho** **^a^** • Increased lifespan [11, 60] • Increased cognition and neuroprotection [49] • Increased glucose induced insulin secretion [32] • Decreased vascular calcification [47] • Nephroprotection [2]	**Effects of low Klotho** **^a^** • Early aging, progenitor cell senescence [44] • Chronic kidney disease^b^ [10] • Increased oxidative stress [57] • Miscarriage^c^ [59] • Placental aging [30] • Growth retardation [56, 58] • Disturbed erythropoiesis [45]
**Klotho can be increased by** • Klotho injection • Aerobic exercise [11] • Losartan [14] • Statins [13] • Fosinopril [14] • Erythropoietin [15]	**Klotho can be decreased by** • Aging [1] • Angiotensin-II [10] • NF-κB signalling [10] • Matrix metalloproteinases [10] • ROS • Chronic kidney disease^b^ [19]

^a^Mice studies.

^b^The relationship between low Klotho levels and chronic kidney disease is bidirectional.

^c^Klotho-deficient mice show early aging traits, but pigs with a monoallelic *Klotho* gene knockout struggle to maintain full-term pregnancies.

NF-κB, nuclear factor κB; ROS, reactive oxygen species.

Thus, the diagnostic use of Klotho in pregnancies is promising, but current evidence is limited by observational character, study size and the lack of standardized analytical methods for the determination of Klotho. Although a sandwich enzyme-linked immunosorbent assay for soluble α-Klotho has been established, different studies utilized different measurement techniques [64]. Additionally, Klotho's role as a biomarker may be influenced by various factors, such as maternal age, gestational age and underlying health conditions [64]. Understanding and accounting for these confounding variables is essential to ensure an accurate interpretation of Klotho levels in relation to foetal health and pregnancy complications. Larger prospective trials and methodological standardization are necessary to establish Klotho as a clinical biomarker for foetal health and pregnancy complications.

## FUTURE PERSPECTIVES

While current evidence is primarily derived from animal studies, translational research is crucial to validate findings in human populations. The novel and yet fragmentary evidence indicating a role of maternal Klotho in placental aging, preeclampsia and preterm delivery emphasizes the need for deeper exploration. Evaluating the potential role of maternal Klotho as a biomarker necessitates extensive prospective studies on a larger scale, considering confounding factors such as maternal age and gestational age. In addition, the origin, biological functions and possible pathophysiological implications of foetal Klotho require further investigation with special consideration of its role in modulating critical pathways like Wnt signalling, potentially affecting oocyte maturation and embryogenesis as well as in organ development. Meanwhile, comprehending the therapeutic potential of Klotho in pregnancy, embryogenesis and foetal development requires a deeper understanding of the mechanisms that underlie its protective properties and investigating the potential of administering it exogenously for conditions such as gestational diabetes, including randomized controlled trials.

## CONCLUSION

**Figure 1:**
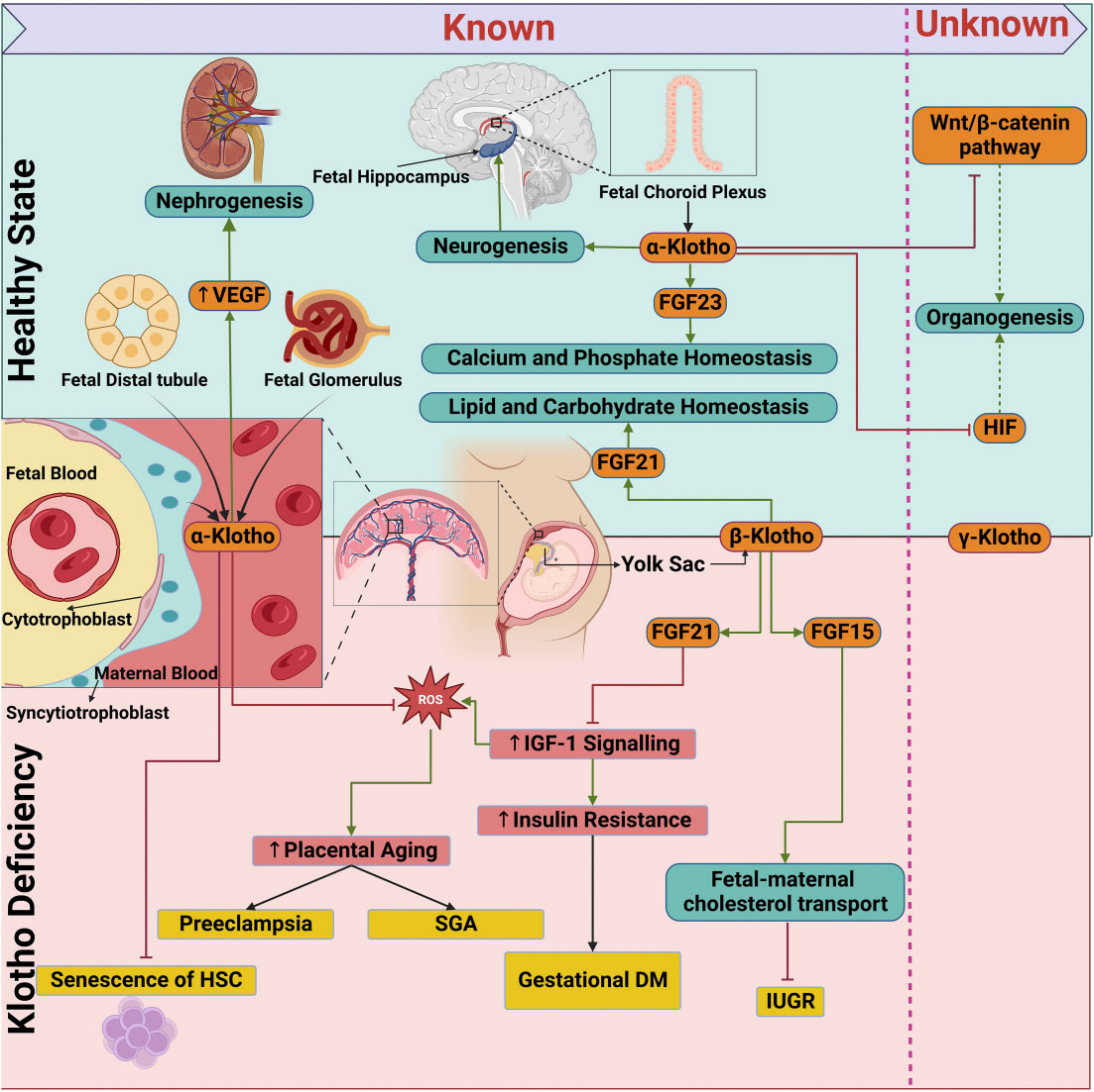
Summary of the molecular basis, physiological and pathophysiological impacts of the Klotho proteins on pregnancy and prenatal development. Klotho's physiological role in prenatal development is summarized in the upper part of the figure. The effects of Klotho deficiency on pregnancy-associated diseases and foetal health are shown in the bottom part of the figure. Potential functions are shown on the right side of the figure. α-Klotho decreases oxidative stress and prevents aging of placenta by suppressing IGF1 signalling. Placental aging disrupts placental circulation and predisposes to pre-eclampsia in mothers and SGA in newborns. α-Klotho is involved in neurogenesis, most notably the development of foetal hippocampus. Moreover, α-Klotho increases VEGF expression and stimulates vasculogenesis and tubulogenesis in the foetal kidney. Additionally, α-Klotho is required for normal haematopoiesis. Through activation of FGF23, α-Klotho controls calcium and phosphate metabolism in intrauterine life. α-Klotho inhibits Wnt/β-catenin and HIF pathways with yet clinically undetermined effects in embryogenesis and organogenesis. β-Klotho is primarily secreted in the yolk sac in the prenatal period. As co-factor to FGF21 and FGF15, β-Klotho helps to maintain lipid and carbohydrate homeostasis and foetal–maternal cholesterol transport. Insufficiency of the β-Klotho–FGF15 axis promotes intrauterine growth restriction.

In conclusion, Klotho, an anti-aging protein, plays a crucial role in the intrauterine period, influencing lifelong health. Therefore, it holds great potential as a biomarker, a therapeutic target and treatment. This review highlights the physiology and pathophysiology of Klotho during intrauterine development, providing a detailed background and indicating possible clinical implications. While current knowledge of the link between Klotho and intrauterine metabolism and organogenesis is promising, it still remains insufficient, encouraging the need for further exploration, including the use of Klotho as a biomarker and therapeutic agent in various pregnancy conditions for preventing foetal complications (Fig. 1).

## Data Availability

Our manuscript has no associated data.
